# Targeted alternative splicing of TAF4: a new strategy for cell reprogramming

**DOI:** 10.1038/srep30852

**Published:** 2016-08-08

**Authors:** Jekaterina Kazantseva, Helle Sadam, Toomas Neuman, Kaia Palm

**Affiliations:** 1 Protobios LLC, Tallinn, Estonia; 2 Cellin Technologies LLC, Tallinn, Estonia; 3 The Department of Gene Technology, Tallinn University of Technology, Tallinn, Estonia

## Abstract

Reprogramming of somatic cells has become a versatile tool for biomedical research and for regenerative medicine. In the current study, we show that manipulating alternative splicing (AS) is a highly potent strategy to produce cells for therapeutic applications. We demonstrate that silencing of hTAF4-TAFH activity of TAF4 converts human facial dermal fibroblasts to melanocyte-like (iMel) cells. iMel cells produce melanin and express microphthalmia-associated transcription factor (MITF) and its target genes at levels comparable to normal melanocytes. Reprogramming of melanoma cells by manipulation with hTAF4-TAFH activity upon TAFH RNAi enforces cell differentiation towards chondrogenic pathway, whereas ectoptic expression of TAF4 results in enhanced multipotency and neural crest-like features in melanoma cells. In both cell states, iMels and cancer cells, hTAF4-TAFH activity controls migration by supporting E- to N-cadherin switches. From our data, we conclude that targeted splicing of hTAF4-TAFH coordinates AS of other TFIID subunits, underscoring the role of TAF4 in synchronised changes of Pol II complex composition essential for efficient cellular reprogramming. Taken together, targeted AS of *TAF4* provides a unique strategy for generation of iMels and recapitulating stages of melanoma progression.

Alternative splicing (AS) is a key process regulating gene expression and underlying proteome diversity. By changing the activity of transcription factors, AS affects cell growth, differentiation^1^,^2^, survival^3^,^4^ and tumourigenesis^5^,^6^,^7^. Changes in the splicing patterns accompany frequently with reprogramming of somatic cells into induced pluripotent stem cells (iPSCs)^8^,^9^,^10^. The discovery of methods for generation of iPSCs by use of specific transcription factors, chromatin-modifying compounds, non-coding RNAs and low molecular weight substances has provided different promising strategies for development of tools for different disease modelling and cell therapy applications^11^. The pioneering study of *in vitro* somatic cell reprogramming used forced expression of MyoD to convert mouse fibroblasts into muscle cells^12^. Use of various combinations of lineage-specific transcription factors has become by now a widely acknowledged approach for direct conversion of fibroblasts into functional neurons, hepatocytes, cardiomyocytes and melanocytes^13^,^14^,^15^,^16^. Research on cellular reprogramming is growing at high speed by applying it to numerous target cells and miscellany of reprogramming factors^17^. However, regulated AS as a tool for effective cell reprogramming has not been actively pursued.

It is commonly known that mechanisms of cellular reprogramming share similar features with cancer initiation^18^. For example, pluripotency transcription factors c-MYC and KLF4 are commonly known as proto-oncogenes^19^; similar signalling pathways are active in cancer development and upon generation of iPSCs^18^,^20^. Down-regulation of tumour suppressor genes, such as p53, enhances reprogramming efficiency^21^, while premature termination of cell reprogramming *in vivo* leads to cancer development^22^. It is even speculated that *in vivo* cancer progression could be initiated by reprogramming-like events^23^. Despite all these findings, only a few reports have been able to convincingly demonstrate successful reprogramming of cancer cells^24^.

According to the current view, general transcription machinery is a dynamic and cell context-specific structure^25^. Transcription factor TFIID as a subunit of the general transcription machinery consists of TATA-binding protein (TBP) and up to 14 TBP-associated factors (TAFs)^26^,^27^,^28^. Most of the TAF subunits in TFIID complex are needed for self-renewal of human embryonic stem cells (hESCs)^29^, while a few of TFIID subunits are required for cell differentiation^30^,^31^,^32^. TAF4 is one of the major structural and regulatory components of TFIID. Previous studies have found that *TAF4* is subjected to extensive cell- and tissue-specific splicing^33^,^34^,^35^. Our recent data show that splicing events in the region encoding the co-activator hTAF4-TAFH domain control the differentiation of human neural progenitors (NHNPs)^35^,^36^ and human adipose-derived mesenchymal stem cells (hMSCs)^34^. Targeted proteolysis of Taf4 was demonstrated to be necessary for differentiation of mouse F9 embryonic carcinoma cells^37^ and myogenic differentiation of myoblasts^38^, whereas enforced expression of TAFH domain blocked differentiation of F9 cells towards early endodermal lineages^39^. Moreover, inactivation of *Taf4* in mouse epidermis resulted in hyperplasia and development of aggressive melanomas in the dermis compartment^40^,^41^. In keratinocytes, the absence of *Taf4* led to ectopic expression of melanocyte-specific *melan-A* and melanoma-associated antigen 9 (*MAGEA9*) and development of invasive melanomas^40^. Interestingly, an upstream enhancer containing binding sites for pluripotency transcription factors Oct4 and Nanog was identified in mouse *Taf4* gene^42^. Most recent findings described TAF4 as one of the critical components in converting human fibroblasts into iPSCs^43^. Thus, with important functions in maintenance of TFIID stability and integrity, TAF4 represents a unique tool for manipulating the whole TFIID composition and in promoting specific cellular programs.

Here, we provide a new concept for cell reprogramming, where instead of changing the transcription regulatory networks by forced expression of lineage-specific transcription factors or use of different miRNAs, we advocate for targeted AS of the core components of RNA Pol II transcription machinery. As an example, we targeted the activity of TAF4 by TAFH-specific RNAi to examine the potential of this approach for reprogramming of human dermal fibroblasts. Data presented here allow us to conclude that targeting AS of TAF4 affects the entire TFIID complex, providing a unique model system to induce iMels and reprogram tumour cells to less or more aggressive cancer phenotypes.

## Results

### Differential activity patterns of hTAF4-TAFH are characteristic to dermal fibroblasts, melanocytes and melanoma cells

**Figure 1:**
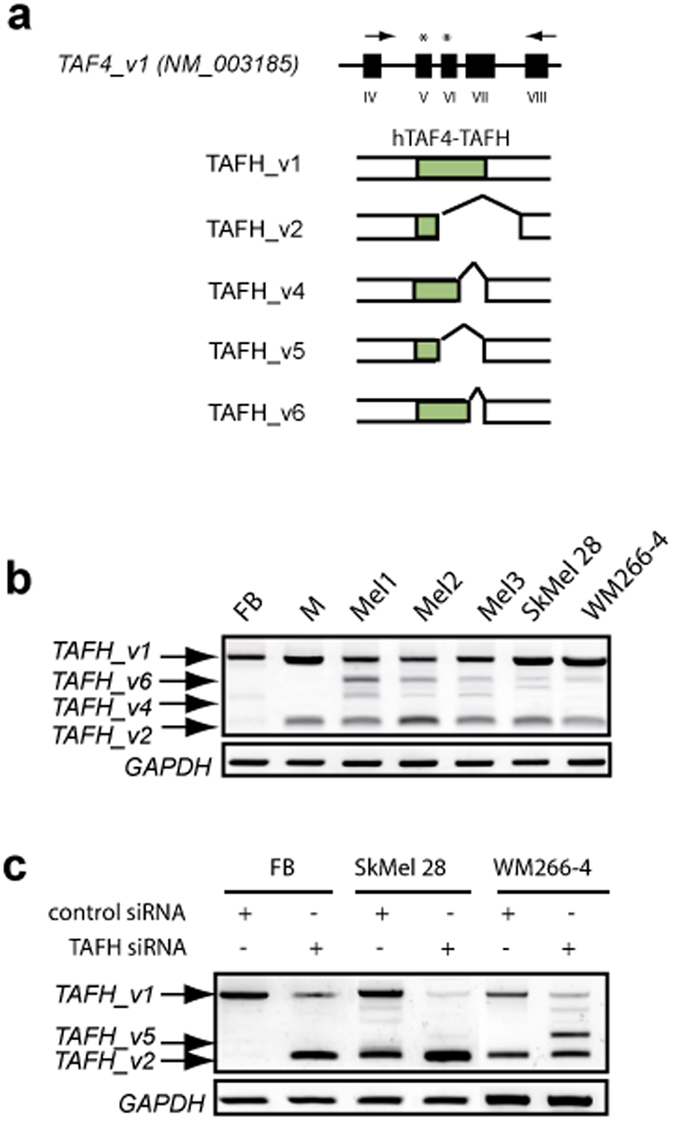
Expression of *TAF4* ASVs in human dermal fibroblasts, melanocytes, primary melanoma and immortalised melanoma cells. (**a**) Schematic representation of a fragment of *TAF4* gene relevant to the current study. Black blocks with numbers indicated below show exons. Arrows indicate the location of TAFH-specific RT-PCR primers and asterisks correspond to the respective TAFH siRNAs. TAF4 isoforms (TAFH_v1, 2, 4, 5, 6) with splicing-affected hTAF4-TAFH domains (in green) are depicted below the gene structure. TAF4 isoforms are referred according to the previously published data^34^. (**b**) RT-PCR analysis of primary dermal fibroblasts (FB), melanocytes (M), primary melanomas (Mel1-3), and melanoma SkMel28 and WM266-4 cells using TAFH-specific primers. Canonical (*TAFH_v1*) and major alternative splice variants (*TAFH_v2, _v4* and _*v6*) are indicated. (**c**) RT-PCR analysis of TAFH (*TAF4*ex5 and *TAF4*ex6) siRNA-treated fibroblasts, SkMel28 and WM266-4 melanoma cells showed effective disruption of exons encoding hTAF4-TAFH domain. *GAPDH* mRNA expression is used as a loading control.

Previously, we have demonstrated that exons encoding the hTAF4-TAFH domain are subjected to extensive AS in hMSCs and NHNP cells^34^,^35^. To assess whether AS of *TAF4* exons V, VI and VII encoding the hTAF4-TAFH (Fig. 1a) is prevailing in cells of neural crest (NC) origin, we examined the expression of *TAF4* ASVs in facial dermal fibroblasts, melanocytes and melanomas.

Using transcript-specific RT-PCR approach, we exclusively examined the splicing of *TAF4* exons encoding hTAF4-TAFH domain. *TAFH_v1* mRNAs encoding intact hTAF4-TAFH were detected in all analysed cells, while melanocytes, primary melanoma tissues and cultured melanoma cells next to *TAFH_v1* mRNAs expressed *TAFH_v2* mRNAs encoding proteins with no hTAF4-TAFH domain at comparable to *TAFH_v1* levels (Fig. 1b). In primary melanoma tumours and cultured cells, *TAFH_v2* was expressed along with *TAFH_v4* and *TAFH_v6,* which all result in isoforms with no hTAF4-TAFH activity (Fig. 1a,b).

**Figure 2:**
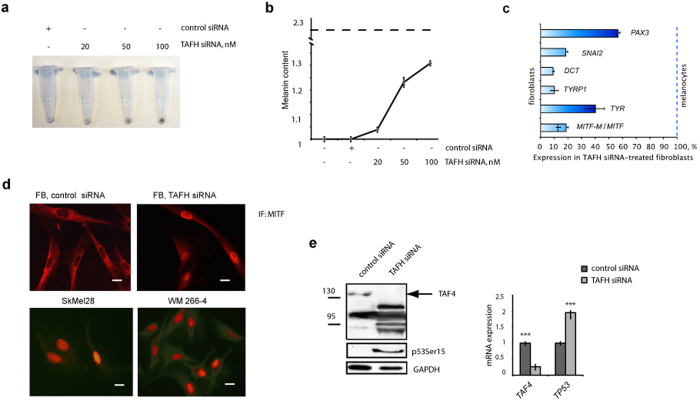
hTAF4-TAFH activity controls conversion of facial dermal fibroblasts to melanocyte-like cells. (**a**) Gradual rise in TAFH siRNA concentration from 20 to 100 nM changed the colour of pelleted fibroblasts to dark brown, indicating to the melanin production in these cells upon treatment. (**b**) Melanin content assay. Fibroblasts were transfected with different amounts of TAFH siRNAs. The relative production of melanin was calculated by measuring cell absorption spectra values at 405 nm in TAFH and control siRNA-treated fibroblasts and normalised to the amount of total protein. For reference control, melanin synthesis was induced by 0.1 mM IBMX (3-isobutyl-1-methylxanthine) in SkMel28 melanoma cells and compared to the value in control siRNA-treated fibroblasts (*dashed line*). (**c**) RT-qPCR analysis showed increased expression of melanocyte-specific genes in TAFH siRNA-transfected fibroblasts relatively to the control siRNA-treated cells at 24 h after treatment. Data shown are relatively to the levels of gene expression in terminally differentiated melanocytes, where 0% is the gene expression in primary fibroblasts, and 100% shows the levels of gene expression in primary melanocytes. Data were received from three independent experiments, with p at least < 0.01. (**d**) Immunofluorescence staining analysis of TAFH or control siRNA-treated fibroblasts at 48 h post-treatment reveals induced expression of MITF in the nuclear compartment of the cells. For positive control, nuclear localisation of MITF expression was verified in SkMel28 and WM266-4 melanoma cells. Scale bar, 40 μm. (**e**) TAFH siRNA-treatment induced up-regulation of p53 in fibroblasts: phosphorylated p53Ser15 (*left*) and *TP53* mRNAs (*right*). mRNA data are normalised to *GAPDH* levels and shown as mean ± SD of three independent experiments with p value < 0.001 (***).

For the detailed analysis of hTAF4-TAFH function, we elaborated RNAi approach with TAFH-specific siRNAs targeting exons V or VI of *TAF4* to affect the expression of different ASVs (Fig. 1a). Upon TAFH RNAi, silencing of *TAFH_v1* mRNAs both in fibroblasts and melanoma SkMel28 and WM 266-4 cells promoted the expression of transcripts encoding isoforms without hTAF4-TAFH (Fig. 1c) that was also verified by changed patterns of TAF4 isoform expression (Fig. 2e). Expression analysis data along with TAFH RNAi intrigued us to study the effects of hTAF4-TAFH activity in NC-derived cells in more detail.

### Silencing of hTAF4-TAFH converts facial dermal fibroblasts to iMels

We have established that abrogation of hTAF4-TAFH activity promotes differentiation of hMSCs and NHNPs^34^,^35^. In the current work, hTAF4-TAFH silencing in fibroblasts by RNAi resulted in highly obvious visual colour change of cell pellets from pale to dark brown in a siRNA dose-dependent manner (Fig. 2a). This finding was in a good agreement with RT-PCR analysis data establishing that expression of *TAF4* ASVs in TAFH RNAi-treated fibroblasts resembled that of melanocytes. Namely, *TAFH_v1* was expressed at low and *TAFH_v2* at high levels (Fig. 1c). Upon TAFH RNAi, the induction of melanogenesis in dermal fibroblasts was further confirmed by the results of melanin content assay (Fig. 2b) and expression analysis of MITF and genes of melanin biosynthesis pathway (Fig. 2c,d). Specifically, treatment of fibroblasts with TAFH-specific siRNAs induced the concentration-dependent increase in the content of melanin relative to the control siRNA-treated cells (Fig. 2b). Conclusively, RT-qPCR analysis of TAFH RNAi-treated fibroblasts compared to the control siRNA-treated cells showed the following (Fig. 2c): a) induced expression of *SNAI2* and *PAX3,* early transcription factors of melanocyte development; b) stimulation of expression of total MITF mRNA and melanocyte-specific alternative form *MITF-M* mRNA; c) increased expression of *MITF* target genes *TYR, TYRP1* and *DCT*. When RT-qPCR data of TAFH RNAi-treated fibroblasts were normalised to the levels of gene expression in primary fibroblasts (arbitrarily referenced as 0%) or melanocytes (taken for 100%), it became apparent that the expression of melanocyte-specific genes in induced melanocytes (iMels) was highly similar to that in melanocytes (Fig. 2c). While immunofluorescence (IF) analysis further confirmed the nuclear expression of MITF in TAFH RNAi-treated fibroblasts similar to melanoma cells (Fig. 2d). Altogether, these data suggested that hTAF4-TAFH activity perturbs the spontaneous conversion of dermal fibroblasts to iMels.

Silencing of hTAF4-TAFH activity in fibroblasts induced the expression of *TP53* mRNAs and accumulation of phospho-p53 (Fig. 2e) similar to the hMSCs^34^, indicating that the function of p53 in the process of hTAF4-TAFH-associated reprogramming is conserved across different cell types.

Altogether, these data argue that TAFH RNAi is an efficient strategy to generate iMel cells from facial dermal fibroblasts.

### hTAF4-TAFH activity restricts differentiation potential of melanoma cells

**Figure 3:**
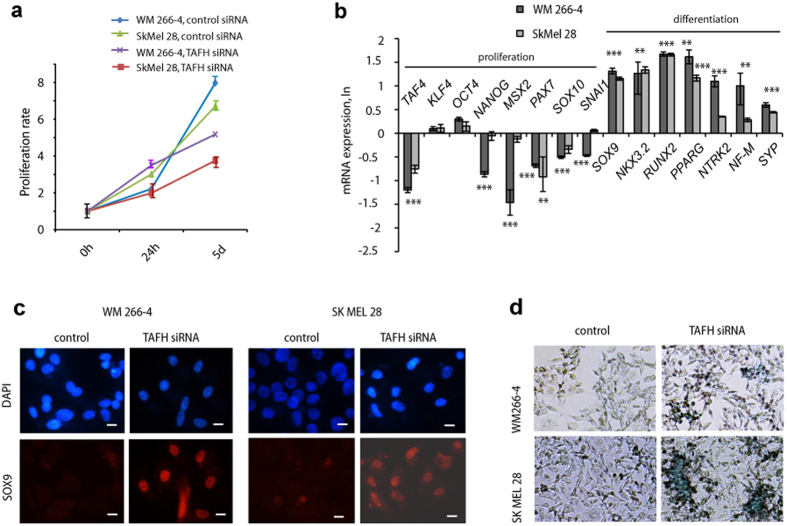
hTAF4-TAFH activity regulates differentiation of melanoma cells. (**a**) Data of WST-1 assay of TAFH and control siRNA-treated WM266-4 and SkMel28 cells subjected to 0 h, 24 h and 5 days of proliferation. In WST-1 proliferation assay, the amount of formazan that occurs during the enzymatic cleavage of the tetrazolium salt WST-1 by cellular mitochondrial dehydrogenases present in viable cells, were measured and normalised to the values in day one of proliferation. The data represented as mean ± SD of three independent experiments. (**b**) RT-qPCR analysis showed that upon TAFH RNAi, the expression of pluripotency-associated (*KLF4, OCT4, NANOG*) and neural crest (*MSX2, PAX7, SOX10, SNAI1*) mRNAs were down-regulated or maintained, while the expression of chondrogenic (*SOX9*, *NKX3.2, RUNX2*), adipogenic (*PPARG*) and neuronal (*NTRK2, MF-M, SYP*) mRNAs were up-regulated in WM266-4 and SkMel28 cells. For differentiation studies, TAFH siRNA-treated for 24 h cells were stimulated to differentiate and analysed at 48 h post-treatment. All data are normalised to *GAPDH* expression and relatively to the expression in control siRNA-treated cells and represented in LN scale. Mean ± SD values from three independent experiments with statistical significance of ***p < 0.001 and **p < 0.01 are shown. Similar statistical findings are indicated with *** in the middle of neighbouring bars. (**c**) Immunofluorescence staining of chondrocyte-specific SOX9 (red) in TAFH or control siRNA-treated WM266-4 and SkMel28 melanoma cells stimulated to chondrogenesis is shown at 7 day of differentiation. Nuclei are stained blue with DAPI (4′, 6-diamidino-2-phenylindole). Results are shown at 20× magnification. (**d**) Alcian blue staining of TAFH or control siRNA-treated WM266-4 and SkMel28 melanoma cells that were stimulated to chondrogenic differentiation for 14 days. Images were taken at 10× magnification.

Based on the data from iMel cells, we expected hTAF4-TAFH activity to control the differentiation of melanoma cells. To examine this, we compared TAFH RNAi effects in two metastatic SkMel28 and WM 266-4 melanoma cells. In both cells, TAFH siRNA treatment suppressed cell proliferation by day 5 (Fig. 3a). Moreover, RT-qPCR analysis revealed that hTAF4-TAFH activity was essential for NC- and stem cell-specific gene expression in melanoma cells (Fig. 3b). Upon TAFH RNAi, expression of pluripotency-associated *NANOG* and NC-specific *MSX2, PAX7, SOX10,* and *SNAI1* mRNAs was significantly reduced, whereas the levels of stem cell factors *KLF4* and *OCT4* remained steady (Fig. 3b). These results suggested that depletion of hTAF4-TAFH activity was needed to initiate differentiation of cancer cells. We tested this hypothesis by using culture conditions supporting lineage-specific differentiation. As expected, silencing of hTAF4-TAFH activity in melanoma cells promoted the initiation of mixed routes of differentiation towards chondrogenic, adipogenic and neurogenic lineages. In differentiation-supported conditions, TAFH RNAi-treated melanoma cells started to express chondrogenic-specific *SOX9*, *NKX3.2* and *RUNX2*; adipogenic *PPARG*; and neurogenic *NTRK2*, *NF-M* and *SYP* mRNAs at 48 h post-stimulation of differentiation (Fig. 3b).

Our recent findings established that silencing of hTAF4-TAFH specifically supported the differentiation of hMSCs along chondrogenic lineages^34^. Considering this and given that in general the cartilaginous differentiation of melanomas is regarded to be a rare event^44^,^45^, we decided to analyse TAFH siRNA-treatment effects on melanoma differentiation in chondrogenesis-supporting media conditions in more detail. Immunoanalysis data revealed that at 7 days of differentiation, TAFH RNAi-treated melanomas showed high levels of expression of chondrocyte-specific SOX9 proteins (Fig. 3c) and prominent staining with Alcian blue that evidenced the synthesis of cartilage-specific ECM-proteins (Fig. 3d). All these data further argued that hTAF4-TAFH rendered TAF4 to control chondrogenic differentiation in melanoma cells.

In sum, TAFH RNAi strategy was beneficial for reprogramming of melanoma cells to recapitulate different stages of cancer progression and unmask the differentiation potential of melanomas along lineages supported by the environmental surrounding.

### hTAF4-TAFH controls migration of iMels and invasiveness of melanoma cells

**Figure 4:**
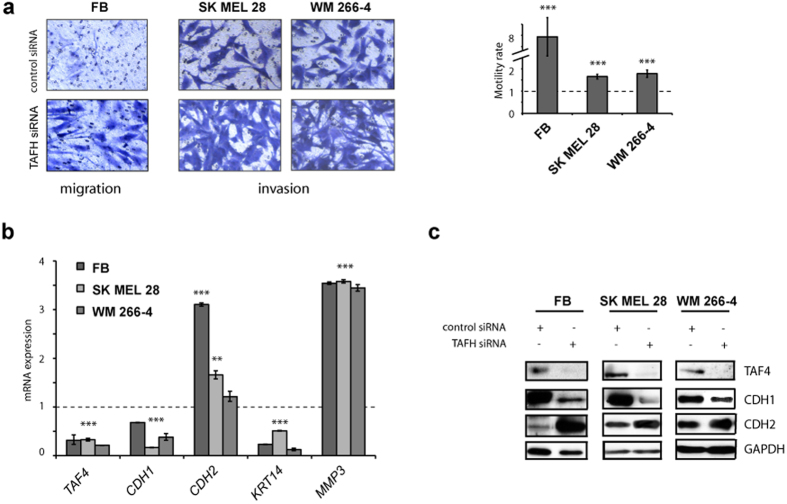
hTAF4-TAFH activity controls expression of E- and N-cadherins and cell motility of dermal fibroblasts and melanoma cells. (**a**) Cell migration and invasion assays showed that TAFH RNAi accelerated the motility of fibroblasts and SkMel28 and WM266-4 melanoma cells. Control and TAFH siRNA-treated cells were grown for 72 h following serum-starvation for 16 h before induction of migration. Migrated towards 10% FBS as a chemoattractant cells were stained with 0.1% crystal violet and photographed (10× magnification). The number of migrated cells was determined by counting from five independent microscopic fields and represented as mean ± SD with ***p < 0.001. (**b**) Relative expression of indicated genes (*TAF4, CDH1, CDH2, KRT14* and *MMP3*) in dermal fibroblasts and melanoma cells after treatments with TAFH siRNAs as analysed by RT-qPCR and compared to control siRNA treatments. Dashed line shows basal levels of gene expression in control siRNA-treated cells. At least three independent experiments were performed and represented as mean ± SD with **p < 0.01 and ***p < 0.001. (**c**) Western blot analysis of total protein extracts showed changes in the expression of CDH1 (E-cadherin) and CDH2 (N-cadherin) upon TAFH RNAi as compared with control siRNA-treated cells. Equal loading was confirmed by GAPDH expression.

Given that differentiation is accompanied by enhanced migratory activity of lineage-committed cells, we analysed the effects of TAFH RNAi on cell motility. For that, migration and invasion potential of iMels and melanoma cells was evaluated by measuring their migration activity towards 10% FBS across polycarbonate membrane. For invasion experiments, membranes were coated with ECM proteins derived from primary fibroblasts that were initially cultivated on the same transwell membranes. TAFH RNAi induced a robust (7-fold) rise in migration of iMels and also supported the invasion of melanoma cells (Fig. 4a). Consistent with these findings, both, iMels and cancer cells, showed high expression of *CDH2* and matrix metallopeptidase 3 (*MMP3*) mRNAs and low expression of *CDH1* and keratin 14 (*KRT14*) mRNAs at 72 h after TAFH RNAi (Fig. 4b). Western blot analysis further confirmed the induction of expression of N-cadherin (CDH2) and inhibition of E-cadherin (CDH1) upon effective hTAF4-TAFH silencing in both cell types (Fig. 4c).

The results from the migration and invasion assays established that suppression of hTAF4-TAFH activity was critical for high motility of iMels and invasiveness of melanoma cells.

### Enforced expression of TAF4 supports cancer stem cell-like features in melanoma cells

**Figure 5:**
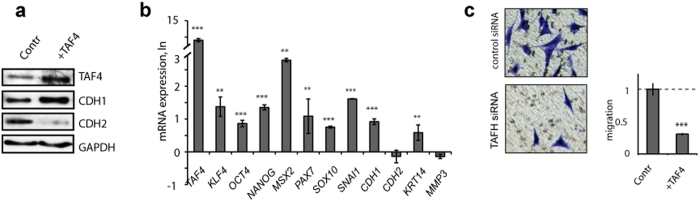
Effects of enforced expression of TAF4 on invasion and multipotency of melanoma cells. (**a**) Western blot analysis of whole cell extracts confirmed the high levels of expression of TAF4 and E-cadherin (CDH1), and low expression of N-cadherin (CDH2) in SkMel28 cells transfected with TAF4 recombinant expression vector at 72 h post-transfection. Equal loading was confirmed by GAPDH expression analysis. (**b**) RT-qPCR analysis of pluripotency, NC (neural crest) and migration-associated genes upon enforced expression of TAF4 in SkMel28 cells showed that high levels of *TAF4* expression support multipotency and inhibit invasion. Data are normalised to *GAPDH* expression relatively to control plasmid-treated cells and represented in LN scale. At least three independent experiments were performed for each analysis and represented as mean±SD with ***p < 0.001, and **p < 0.01. (**c**) Cell invasion assays showed that high levels of TAF4 suppress the invasion potential of SkMel28 cells. The number of invaded cells was determined by counting from five independent microscopic fields, normalised to cell numbers that were transfected with control vector (Contr) and represented as mean±SD with ***p<0.001 determined by Student t-test.

To further assess the role of hTAF4-TAFH activity in cell reprogramming, we investigated whether melanoma cells were amenable to reprogramming towards induced cancer stem cell (iCSC)-like cells by using forced expression of TAF4. Overexpression of TAF4 resulted as expected in the heightened levels of TAF4 protein (Fig. 5a) and significantly increased *TAF4_v1* mRNA expression (Fig. 5b), and brought along phenotypic changes of SkMel28 melanoma towards cells with drastically reduced invasive potential (Fig. 5c). These cells had lost their ability to migrate because of the high hTAF4-TAFH activity that switched cells to predominant expression of E-cadherin (Fig. 5a). These changes were also observed at mRNA levels (Fig. 5b). Consistently, *CDH2* and *MMP3* levels were reduced, and *CDH1* and *KRT14* mRNA levels increased in these cells (Fig. 5b). On the other hand, enforced expression of TAF4 promoted expression of pluripotency- associated *KLF4*, *OCT4* and *NANOG,* and NC-related *MSX2, PAX7, SOX10* and *SNAI1* (Fig. 5b), reaffirming the critical role of hTAF4-TAFH activity in the maintaining of multipotency in melanoma cells.

All in all, data of melanoma cell reprogramming by enforced expression of TAF4 evidenced the fast induction of multipotent features in tumours just by altering the hTAF4-TAFH activity levels in cells.

### TAFH RNAi results in changed splicing of transcripts encoding TFIID complex subunits

**Figure 6:**
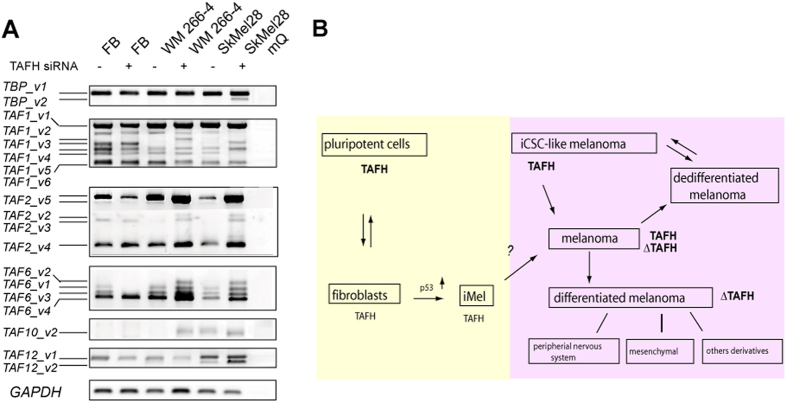
hTAF4-TAFH governs AS events of different TFIID subunits and global differentiation and migratory potential of NC-derived cells. (**A**) RT-PCR analysis of *TBP*, *TAF1*, *TAF2*, *TAF6*, *TAF10* and *TAF12* in response to TAFH RNAi in primary fibroblasts and cultured melanoma WM 266-4 and SkMel28 cells was performed by using transcript-specific primers. Respective ASVs that were sequence verified are denoted on the left and characterised in more detail in Figure S2. (**B**) High levels of proteins with intact hTAF4-TAFH (TAFH) are inherent to the stem and stem-like cells with low capacity to migrate, while abolished hTAF4-TAFH activity (∆TAFH protein isoforms) is characteristic to highly motile cells committed to differentiate. High levels of expression are shown in *bold*. Yellow colour indicates to the expression of TAF4 proteins in normal NC-derived cells (facial fibroblasts and melanocytes), whereas purple area relates to melanoma cells. Data of TAFH overexpression in fibroblasts are taken from^43^. Scheme is adapted from^71^. iMel–induced melanocytes; iCSC-like cells—induced cancer stem cell-like cells.

Finally, we examined whether TAF4-associated cell reprogramming might affect the expression of other members of TAF complex. Firstly, we analyzed the expression of TAF4b, TAF4 paralog, in response to TAF4-TAFH siRNA treatments (Supplementary Figure S1). Western blot (Fig. S1a) and RT-PCR (Fig. S1b) data established that levels of TAF4b remained unaffected upon TAFH silencing. Next, RT-PCR analysis revealed that human fibroblasts and melanoma cells, in general, expressed a wide variation of ASVs encoding TFIID complex subunit proteins (Fig. 6A and data not shown). Transcript-specific RT-PCR analysis showed that many of these had retained ORFs and thus the potential to code different isoforms (Fig. 6A and Supplementary Figure S2). In fibroblasts, silencing of hTAF4-TAFH activity led to the reduced expression of distinct ORF-retaining *TAF2, TAF6* and *TAF12* ASVs. In melanomas, TAFH RNAi induced the differential expression of *TBP, TAF1*, *TAF2*, *TAF6*, *TAF10,* and *TAF12* ASVs. More specifically, TAFH RNAi in melanoma cells led to the high levels of *TAF6_v3* encoding the protein isoform with altered histone fold domains (HFD) similar to the apoptosis-dependent form of TAF6, namely TAF6δ^46^ (Fig. 6a and Supplementary Figure S2). Furthermore, in SkMel28 melanoma cells, TAFH RNAi promoted the expression of *TBP_v2*, which encodes protein isoforms with no poly-glutamine Armadillo-like helical region that is functionally important for hetero-oligomeric interactions^47^ (Fig. 6a and Supplementary Figure S2). Splicing of TAF1, the largest and multifunctional TFIID subunit, was also affected by perturbations in TAFH structure, introducing alterations into its protein kinase and histone acetyltransferase domains (Fig. 6a and Supplementary Figure S2). Altogether, these data argued for the tight control of TFIID composition and function at the level of AS of *TAF4*. However, yet more data is needed to draw more definite conclusions.

In whole, our data demonstrated for the first time that subunits of TFIID complex were targets of extensive cell-specific AS. Changes in hTAF4-TAFH activity affected the expression of TFIID complex components as a part of the molecular control of cellular reprogramming and cancer development.

## Discussion

This study concludes that splicing patterns retaining high levels of hTAF4-TAFH activity lead to cell phenotypes with high multipotency but low capacity to migrate, while splicing events resulting in low hTAF4-TAFH activity enforce cells to migrate and differentiate (Fig. 6B). Simultaneous presence of TAF4 isoforms with high and low hTAF4-TAFH activities in normal cells and in cancer suggests that dominance of any of these forms is likely to have dramatic effects upon development and in tumour progression.

Current cell separation and cultivation methods have been limiting the use of melanocytes in clinics^48^. Generation of functional melanocytes from a highly accessible cell source such as dermal fibroblasts by cell reprogramming method as described herein would establish an easily scalable supply of autologous melanocytes, which could be beneficial for developing cell-based treatments of congenital pigment disorders, or in the studies of the etiopathogenesis of melanomas. Previous data have substantiated that reprogramming of dermal fibroblasts into functional melanocytes implies the use of certain combinations of exogenously added factors including transcription factors MITF, SOX10 and PAX3^16^. Instead of changing the transcriptional networks by enforced expression of specific transcription factors or microRNAs, this study provides a new concept for manipulating cell fate by targeted AS of general transcription machinery components. Here we show that targeted splicing of exons encoding hTAF4-TAFH domain alters TAF4 activity and converts fibroblasts directly into iMel cells. This is the first study to demonstrate that TAFH RNAi approach is an effective tool for generating the iMel cells with characteristics of normal melanocytes. Moreover, our findings establish a link between hTAF4-TAFH activity and activation of p53 to accompany with effective reprogramming of fibroblasts into iMels. Involvement of p53 in tanning response^49^ and hyperpigmentation of epidermis has been described^50^. Simultaneous activity of MITF and p53 in pigmented skin has suggested a model of crosstalk between these two cellular pathways as necessary for protection of skin against UV harms^49^. Thus, TAF4-p53 interactions may be crucial for effective reprogramming of cells. However, this hypothesis remains yet to be tested.

Recent studies along with ours have demonstrated that differentiated cells do not express canonical TAF4 protein at high levels^30^,^38^,^51^. Herein we show that malignantly transformed cells, melanoma in particular, represent a unique model where both, canonical and alternative TAF4 isoforms, are expressed at comparable levels providing an attractive context for manipulating cancer cells *in vitro*. Similar to hMSCs^34^, neural progenitor cells^35^, and facial dermal fibroblasts, silencing of hTAF4-TAFH activity in melanoma promotes these cells to differentiate. Melanoma is a highly divergent neoplasia, with prominent genetic and clonal heterogeneity^52^,^53^. The phenomenon of reversal differentiation is well described for NC-derived lineages^54^,^55^. In the current study, we have demonstrated the critical role of hTAF4-TAFH in driving the fibroblast-like (mesenchymal) features in melanoma cells, as silencing of hTAF4-TAFH activity promoted melanoma cells to differentiate along mesenchymal lineages. In addition, enforced expression of hTAF4-TAFH revealed that melanoma cells avidly acquire phenotypes of highly plastic NC stem cell-like cells^56^ and characteristics of iCSCs^57^,^58^, supporting the expression of pluripotency *KLF4*, *NANOG* and *OCT4* and genes associated with multipotent NC phenotype. It is highly conceivable that similar changes in AS of *TAF4* with consequences to reprogramming of cancer cells can spontaneously occur *in vivo.* In sum, our study is the first to pinpoint to the role of hTAF4-TAFH activity of TAF4 as one of the mechanisms in driving heterogeneity in melanoma, where cells with low hTAF4-TAFH activity differentiate along different lineages and cells with high activity of hTAF4-TAFH retain multipotency.

Our current data suggest that integrity of hTAF4-TAFH domain is critical for cell migration. We established that changes in the activity of hTAF4-TAFH can spontaneously convert dermal fibroblasts into highly motile iMels and support invasion of melanoma cells. Low levels of hTAF4-TAFH activity induce E- to N-cadherin switches in these cells. Changes in the expression of these ECM proteins are a characteristic of epithelial-to-mesenchymal transition (EMT), which is a crucial process in development and in cancer progression^59^. Current findings provide a new understanding of how one of the core processes of EMT is under the control of hTAF4-TAFH activity.

By analysing the expression of ASVs of different subunits of TFIID in response to TAFH RNAi, we found that cell-specific AS is highly common for components of TFIID complex. The majority of TAF subunits are expressed in tissue- and development-specific manner. The functions of TAF4, TAF8 and TAF10 have been found to be critical for maintaining pluripotency^43^, whereas TAF2, TAF7 and TAF12 have come across as ESC determinants^60^. Silencing of TAF3 and TAF5, on the other hand, results in loss of OCT4 function^61^,^62^. Differential expression of TAF subunits drives lineage-specific differentiation^29^. For example, TAF3 is predominantly expressed in myocytes^38^; expression of TAF1, TAF3, TAF4 and TAF9 is absent in liver^63^; TAF8 is essential for adipogenic but not for myogenic differentiation^64^. Our data extend these findings by showing that TAF isoforms generated by cell-specific AS control the composition and activity of cellular TFIID complexes through dynamic regulation of their tissue- and development-specific functions. Flexible arrangement of TFIID complexes in different cellular contexts offers an efficient means to control cell fate by targeted AS of a single component of TFIID, such as established by us TAF4 subunit. Previous studies have shown that TAF4b can compensate for the loss of TAF4 at early stages of embryogenesis^65^, consistent with the earlier findings that both proteins retain specific and non-redundant functions^66^. Thereby, although TAF4 and TAF4b have at some extent overlapping roles, part of their functions is specific. As TAF4-TAFH-dependent mechanism of cell reprogramming is clearly not associated with the compensatory functions of TAF4b, this suggests that alternative splicing of TAF4 leads to TAFH-less protein isoforms that lack the ability to substitute for canonical TAF4 activity in RNA PolII-dependent transcription, but behave as dynamic fine-tuners of specific cell differentiation programs dictated by the surrounding.

In conclusion, a more detailed understanding is required to clarify the role of TAF4 in the sequence of events driving the generation of iMels and cancer progression. However, it is quite clear from our studies that targeted AS of *TAF4* could be used as a new way for cell reprogramming with advantages in cell-based therapies.

## Materials and Methods

### Ethics Statement

Use of human biological materials for the study was pre-approved by the local ethical committee at the National Institute for Health Development, Tallinn, Estonia (Approval No 2234 from Dec 09, 2010). All experiments were performed in accordance with relevant guidelines and regulations. Written informed consent was obtained from all participants prior to the study.

### Cell culture

Human primary dermal fibroblasts were freshly isolated from the facial eye-lid skin of women aged between 30 to 50 years^67^, expressed typical for fibroblasts specific cell-surface markers CD73, CD90, CD105, and were negative of CD45 by analysis of flow cytometry as previously described^68^. All fibroblasts expressed NC markers including *SLUG/SNAIL*, *SOX9*, *SOX2*, *PAX3*, *PAX7* and *MSX1* (Supplementary Table S3). The cells were propagated in the medium of low glucose DMEM with glutamine (DMDM-LG) (PAA Laboratories, Austria) supplemented with 1% penicillin/streptomycin (PAA Laboratories, Austria) and 10% heat-inactivated foetal bovine serum (FBS) (PAA Laboratories, Austria) in a humidified atmosphere at 37 °C and 5% CO_2_. Throughout the study, fibroblasts from low (<7) number passages were used in the functional assays. WM266-4 and SkMel28 human melanoma cell lines were obtained from ATCC and cultured in DMEM high glucose medium with glutamine (DMEM-HG)(PAA Laboratories, Austria) supplemented with 10% FBS and 1% penicillin/streptomycin.

### Transfection

Fibroblasts and melanoma cells were treated with TAFH-specific siRNAs (5′-GGUUAUACCGAGAACUUAA-dTdT-3′ and 5′-CAGCUAAUGUGAAAGAGCU-dTdT-3′ referred here as *TAF4ex5* siRNA and *TAF4ex6* siRNA correspondently) as described^34^. All experiments were performed with both siRNAs, but in case of similar results obtained, siRNAs are designated in Figures as TAFH siRNAs. All synthetic Silencer^®^ Pre-designed siRNAs together with Negative Control #2 siRNAs were purchased from Ambion, Invitrogen (UK). Cultured cells were transfected with 25 or 50 nM of siRNAs using Lipofectamin RNAiMAX reagent (Invitrogen, UK) according to the manufacturer’s protocol. For over-expression studies, 5 × 10^5^ melanoma cells were transfected with 0.5 μg of full-length *TAF4* cDNA in pcDREAM2.1 (GenScript, Piscataway, NJ, USA) using Lipofectamine 3000 (Life Technologies). pmaxGFP^®^ vector was used for transfection control. Cells were harvested at indicated time points and used in functional assays.

### RNA isolation, RT-PCR and Real-Time PCR

Human primary melanoma and normal tissue RNA panels were purchased from BioChain Institute (USA). For cultured cells, total RNA was purified using TRIzol^®^ reagent (Invitrogen, Life Technologies, UK) following manufacturer’s recommendations. cDNA was synthesised from DNase-treated (Ambion, UK) RNA using Superscript III RT (Invitrogen, UK) and a mix of oligodT and random hexamers, according to manufacturer’s recommendations. RT-PCR was carried out using HOT FIREpol^®^ Master Mix (Solis Biodyne, Estonia). RT-qPCR was performed in triplicates using LightCycler^®^ SYBR Green I Master Mix (Roche Applied Science) and the LightCycler^®^ 480 Real-Time PCR System (Roche Applied Science). The fold of change was calculated relatively to control siRNA treatments after normalization to *GAPDH* expression, using 2^−ΔΔCt^ method, where ΔCt is (gene of interest Ct) − (GAPDH Ct), and ΔΔCt is (ΔCt treated)—(ΔCt control). Primer sequences are listed in Supplementary Table S4.

### Immunofluorescence and immunoblot analyses

TAFH siRNA-treated and untreated human dermal primary fibroblasts and melanoma cells were grown on 22-mm^2^ glass slides to about 70% confluence. Anti-MITF (ab59232, Abcam) as primary, and Alexa Fluor 546 (Molecular Probes, Invitrogen, Eugene, OR, USA) as secondary antibodies were used for immunofluorescence analysis. Images were visualised using Nikon Eclipse 80i fluorescence microscope (Nikon Instruments Inc., USA).

For Western blot analysis, treated and untreated cells were collected by trypsin-EDTA (PAA Laboratories, Austria). Cell fractionation was carried out using NE-PER Nuclear and Cytoplasm Extraction Reagents (Thermo Scientific, Pierce, Rockford, IL, USA). Total protein concentration of nuclear and whole cell extracts was measured using BCA Protein Assay kit (Thermo Scientific, Pierce, Rockford, IL, USA). The following primary antibodies were used: anti-TAF4 (BD Biosciences, 612054), anti-CDH1 (Abcam, ab53033), anti-CDH2 (Abcam, ab12221), anti-phospho-TP53^Ser15^ (Cell Signaling, 9284), and anti-GAPDH (Sigma, G8795). Secondary HRP-conjugated antibodies were purchased from Abcam. Proteins were visualised using SuperSignal West Dura Chemiluminescent Substrate (Thermo Scientific, Pierce, Rockford, IL, USA).

### Melanin content assay

Melanin content was determined according to the modified method of Hosoi *et al.*^69^. Human dermal fibroblasts were cultured and subjected to mock or siRNA treatments. Melanoma SkMel28 cells were treated with 1 mM dibutyryl-cAMP for 6 days as a control for melanin synthesis. After 24 h, cells were washed by PBS, harvested by trypsinisation and counted. The cell pellet was solubilised in 200 μl of 0.2 M NaOH containing 10% DMSO per 1 × 10^6^ cells at 80 °C for 1 h. The absorbance of each well was measured at 405 nm using SPECTRAmax 340 PC spectrophotometer (Molecular Devises, USA) and the values were normalised to the total amount of protein.

### Proliferation assay

WM266-4 and SkMel28 melanoma cells were grown in 96-well flat bottom tissue culture plates up to 80% confluence and treated with control or TAFH siRNAs. WST-1 analysis (Roche Applied Science) was performed according to manufacturer’s instructions. Absorption at 450 nm was measured using SPECTRAmax 340 PC Microplate Reader (Molecular Devises LLC, USA). The data were analysed using Softmax Pro 3.12 software and normalised to the values at the day of plating. Cell viability was evaluated every 24 h, up to 5 days post-treatment.

### Differentiation

For melanoma differentiation studies, 24 h after siRNA transfection, cells were cultured in conditions supporting adipogenic, chondrogenic or neuronal differentiation as previously described^34^,^35^. The extent of chondrogenic, adipogenic and neuronal differentiation was analysed by RT-qPCR of lineage-specific marker genes. To confirm effective chondrogenic differentiation, immunofluorescence detection by chondrocyte-specific SOX9 (AB5535, Millipore) was performed 7 days after culturing of siRNA-treated melanoma cells in chondrogenic differentiation-supporting conditions. Alcian blue staining (Sigma) was performed 2 weeks after differentiation stimulation. Cells were fixed in 4% PFA, washed with distilled water, stained with Alcian blue and analysed under 10× magnification for glycosaminoglycan expression.

### Cell migration and invasion assays

Cell migration and invasion were analysed by *in vitro* migration assays, using 8 μm-pore polycarbonate transwell filters (QCM^TM^ Colorimetric Cell Migration Assay, Millipore, USA). For melanoma invasion assay, transwell filters were coated with extracellular matrix components (ECM) extracted from human fibroblasts^70^. For that, fibroblasts were plated onto transwell filters, cultivated until the confluence, and ECM were extracted by using cell lysis buffer (10 mM TRIS-HCl, 1 mM EDTA, at pH7.4) for 16 h. For migration assay, transwell filters were left uncoated. For migration/invasion studies, fibroblasts or melanoma cells were treated with TAFH siRNAs for 72 h and thereafter starved in serum-free medium for 16 h. Cells were seeded at density of 1 × 10^5^ cells per insert in DMEM-LG medium without the addition of FBS, whereas the bottom chamber was filled with DMEM-LG supplemented with 10% FBS. The total number of cells that migrated to the lower chamber was quantified after 24 h of incubation of cells at 37 °C with 5% CO_2_. Migrated cells on the lower surface of the filter membrane were fixed and stained by cell stain solution with 0.1% crystal violet before counting. The number of migrated and invaded cells per well was counted in five randomly selected microscope fields in three independent experiments using Nikon Eclipse 80i fluorescence microscope (Nikon Instruments Inc., USA).

### Statistical analysis

At least three independent replicates were performed for each experiment. Statistical analysis was performed using an unpaired Student’s *t*-test with a 2-tailed *p* value. Differences were considered significant when the *p* < 0.05.

## Additional Information

**How to cite this article**: Kazantseva, J. *et al.* Targeted alternative splicing of TAF4: a new strategy for cell reprogramming. *Sci. Rep.* **6**, 30852; doi: 10.1038/srep30852 (2016).

## Supplementary Material

Supplementary Information
