# Supplementary material for: Targeted alternative splicing of TAF4: a new strategy for cell reprogramming
Source: Sci Rep. 2016 Aug 8;6:30852. doi: 10.1038/srep30852 (PMC4976350; doi:10.1038/srep30852)
Supplement: Supplementary Information [file srep30852-s1.docx]

**Targeted alternative splicing of TAF4: a new strategy for cell reprogramming**

Jekaterina Kazantseva, Helle Sadam, Toomas Neuman, and Kaia Palm

**Supplementary Information**

**S1 Figure. Expression of TAF4b in response to TAF4-TAFH silencing**

**
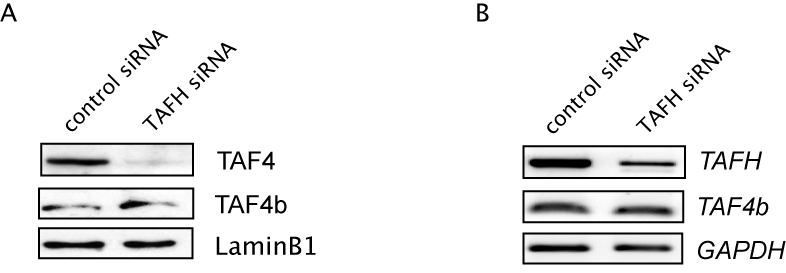
**

**Expression of TAF4b does not change in response to TAF4-TAFH RNAi in fibroblasts.** Western blot of nuclear extracts from control and TAFH siRNA treated fibroblasts (A) and RT-PCR (B) analyses demonstrated that levels of TAF4b are not affected upon TAF4-TAFH silencing. For immunoblot detection, TAF4b antibodies (Santa Cruz, SC-85178) were used. Equal loadings are represented by LaminB1 and *GAPDH* levels.

**S2 Figure. Schematic representation of protein structure and exonic composition of TAF complex subunits.**

The simplified protein structures of TBP, TAF1, 2, 6, 10 and 12 are provided. Black boxes are related to exonic insertions, while triangles represent exonic deletions. Coloured ovals demark functional domains: His-rich – Histidine-rich motif; HFD – histone-fold domain; PK – protein kinase domain; HAT – hystone acetyltransferase domain; NLS – nuclear localization signal; ARM – Armadillo repeat motif. Schematic representations of exon structure where exon numbers and positions of transcript-specific primers (arrows) are indicated below the each protein structure. These transcripts correspond to data in Figure 6A and have been sequence verified.

**S3 Table. Ct values of expression of NC-specific mRNAs in facial dermal fibroblasts**

| Gene name | Average Ct value (if Ct of GAPDH is 17) | StDev |
| --- | --- | --- |
| ***SNAIL1*** | 22.49 | 0.83 |
| ***SLUG*** | 21.16 | 0.54 |
| ***SOX9*** | 32.11 | 1.57 |
| ***SOX2*** | 24.58 | 0.34 |
| ***PAX3*** | 23.35 23.35179143 | 0.70 |
| ***PAX7*** | 24.89 | 1.39 |
| ***MSX1*** | 19.25 | 0.59 |
| ***MSX2*** | 28.33 | 1.25 |

**S4 Table. List of primers**

| Gene | Sense primer | Antisense primer |
| --- | --- | --- |
| *TAF4* TAFH | CTGCCACGGAAACTATGGAA | CTTTACAGGACCGCGTTAGC |
| *TAF4 Real* | AGAAACAGCTCAGCAGAAGAAC | GCCTGCTCATATCTGTCGTCAT |
| *TBP* | CGGCTGTTTAACTTCGCTTC | TTGCAGCTGCGGTACAATCC |
| *TAF1* | AGACACGGACAGCGACGAA | ACCACCTGAAGCTTGCCTC |
| *TAF2_1* | CCACTAGAACCTGGTCAAATAC | GACTGAGAGTGGAGCGCTTG |
| *TAF2_2* | GGCTTATGTTGAAGTGGCTG | AGCAGTACTAGCCAGACTTAG |
| *TAF5* | GGAGGAGCAGACGGAGGT | CGTCATCCAAAGGTACCTCAA |
| *TAF6* | ACCCTTTGAAGGTCCCTTGTG | TAGAGCTGCTGCTCCACAGAC |
| *TAF10* | TCTCCCACCGGCCCGATGA | GCAAACACAGAAGAGCGACTAG |
| *TAF12* | CTTTAATTGGCCTTGGTCCTC | TGTTTGTGAGCTTCTGTGGTG |
| *TP53* | TGGAGGAGCCGCAGTCAGATCC | TTGCTTGGGACGGCAAGGGG |
| *MITF* | GGCTTGATGGATCCTGCTTTGC | GAAGGTTGGCTGGACAGGAGTT |
| *MITF-M Real* | TTATAGTACCTTCTCTTTGCC | GCTTGCTGTATGTGGTACTTG |
| *TYR* | GCACAGATGAGTACATGGGAGG | CTGATGGCTGTTGTACTCCTCC |
| *TYRP1* | TCTCAATGGCGAGTGGTCTGTG | CCTGTGGTTCAGGAAGACGTTG |
| *DCT* | GACCTGCATTTGTTACCTGGC | CCAGTGGCAAAGTTCCAGTAG |
| *SLUG* | ATACCACAACCAGAGATCCTCA | GACTCACTCGCCCCAAAGATG |
| *PAX3* | CCGAGACAAATTACTCAAGGACG | GCTGATGGAACTCACTGACGG |
| *SOX2* | GAAGAAGGATAAGTACACGCTGC | GTTCATGTGCGCGTAACTGT |
| *SOX10* | CTTCATGGTGTGGGCTCAG | GTGGTCTTTCTTGTGCTGCA |
| *CDH1* | GAATCCAAAGCCTCAGGTCA | CCCACCTCTAAGGCCATCT |
| *CDH2* | ATCGTGTCTCAGGCTCCAAG | GGATTGCCTTCCATGTCTGT |
| *KRT14* | TGCAGATTGAGAGCCTGAAG | ATTGACATCTCCACCCACCT |
| *MMP3* | GCATCCACACCCTAGGTTTC | AATCCCTGGAAAGTCTTCAGC |
| *KLF4* | CACCCACACTTGTGATTACGC | GTGTGTTTACGGTAGTGCCTG |
| *OCT4* | AAGCGAACCAGTATCGAGAACCG | TCGCTGCTTGATCGCTTGCCC |
| *NANOG* | GGTGTGACGCAGAAGGCCTCA | AGTCGGGTTCACCAGGCATCC |
| *NKX3.2* | CCGCTTCCAAAGACCTAGAGGA | ACCGTCGTCCTCGGTCCTTGG |
| *RUNX2* | TAACTTGTGGCTGTTGTGATGCG | CTCACGTCGCTCATCTTGC |
| *PPARG* | TGCAGTGGGGATGTCTCATA | CTGGATTCAGCTGGTCGATA |
| *NTRK2* | TGATGATGACTCTGCCAGCCC | TTGAGCTGACTGTTGGTGATGCCA |
| *NF-M* | GAGTGGTTCAAATGCCGCTAC | CTCTAGCTCGATGCTCTTGGA |
| *SYP* | AGTTGGGGACTACTCCTCGTC | GGCCCTTTGTTATTCTCTCGGTA |
| *GAPDH* | GAAGGTGAAGGTCGGAGT | GCATGGACTGTGGTCATGAG |
| *GAPDH Real* | CTCTCTGCTCCTCCTGTTCGAC | TGAGCGATGTGGCTCGGCT |
